# PowerPlex® fusion 6C system: internal validation study

**DOI:** 10.1080/20961790.2018.1430471

**Published:** 2018-02-09

**Authors:** Ana Boavida, Vanessa Bogas, Lisa Sampaio, Nair Gouveia, Maria J. Porto, Francisco Corte-Real

**Affiliations:** Forensic Genetic Service, Centre Branch, National Institute of Legal Medicine and Forensic Sciences,Coimbra, Portugal

**Keywords:** Forensic science, forensic genetics, PowerPlex® Fusion 6C, amplification, short tandem repeat sequence (STRs), validation

## Abstract

This work is aimed at describing the proceedings and parameters used to validate PowerPlex® Fusion 6C System, the polymerase chain reaction (PCR) amplification kit by Promega, for posterior implementation in the laboratorial routine of the Forensic Genetic Service. The PowerPlex® Fusion 6C System allows multiplex PCR, through simultaneous amplification and posterior detection by fluorescence of 27 loci. Characterization of the kit was made according to the laboratory's internal validation procedure based on validation guidelines from Scientific Working Group on DNA Analysis Methods. Some parameters were evaluated, such as specificity, analytical thresholds, sensitivity, precision, mixture studies, DNA control samples, a proficiency test and changes in the PCR-based procedures: final reaction volume and cycle number, changes in the reaction mixture for direct amplification. This kit proved to be very robust and the results are in concordance with previous developmental validation by the manufacturer. In some parameters, the results were better than expected.

## Introduction

PowerPlex® Fusion 6C System, by Promega, is one of the most recent polymerase chain reaction (PCR) amplification kits available for human identification with a higher discriminatory power. It also enables direct amplifications from FTA® Card punches and from swabs. This kit has a six-dye multiplex system and allows the amplification and detection by fluorescence of the 20 autosomal loci in the expanded Combined DNA Index System (CODIS) core loci [1] (*CSF1PO, D3S1358, D5S818, D7S820, D8S1179, D13S317, D16S539, D18S51, D21S11, FGA, TH01, TPOX, vWA, D1S1656, D2S441, D2S1338, D10S1248, D12S391, D19S433* and *D22S1045*), as well as Amelogenin for gender determination. The loci *Penta D, Penta E* and *SE33* are also included, and three exclusive Y chromosome alleles (*DYS391, DYS576* and *DYS570*), allowing allelic attribution in a total of 27 loci. This extension of genetic markers satisfies both CODIS and European Standard Set recommendations [1,2].

Despite the existence of a developmental validation [3], each laboratory must do their own validation studies to check if other methodologies, equipments, storage conditions and workflow are compatible with the new kit before it is introduced in the laboratorial routine. All parameters should be evaluated to know the abilities and efficiency of the kit.

This work describes the validation studies conducted in the Forensic Genetic Service, and the characterization and validation of the kit was performed according to the laboratory's internal validation procedure based on validation guidelines from Scientific Working Group on DNA Analysis Methods [4]. Studies of specificity, analytical threshold (AT), sensitivity, precision, mixtures and a proficiency test were performed. Some changes to the original protocol such as volume reduction and cycle number adaptations and a qualitative study were also made.

Based on this study, an internal protocol was also elaborated, with the necessary adaptations, that can give the best results, to be used when amplifying samples with this kit.

## Materials and methods

### Sample collection

Blood and saliva was collected from 12 unrelated volunteers after informed and written consent was given. Twenty-four stains of blood and saliva were made, 12 stains of each biological fluid and all samples were anonymized. Saliva stains were made after collecting epithelial buccal cells with 4N6FLOQSwabs™ Regular (Thermo Fisher Scientific, MA, USA) then impregnating the cells into an Indicating FTA™ Mini Card (Thermo Fisher Scientific, MA, USA).

Blood stains were collected using a sterile puncture device Accu-Chek® Safe T-Pro® Plus, to make a slight puncture in a finger and then impregnated into the Human ID Bloodstain Card (Thermo Fisher Scientific, MA, USA).

Finally, buccal swabs with Omni Swab (Thermo Fisher Scientific, MA, USA) were also collected from all the volunteers.

### DNA extraction – sample preparation

DNA was extracted/prepared from the samples using different methodologies such as (1) Chelex®100, (2) Prep-n-Go™ buffer and (3) PrepFiler™ Express.

(1) Chelex® 100 extraction was performed according to the protocol described by Walsh et al. [5].
(2) Samples were prepared with Prep-n-Go™ by placing a fragment of the buccal swab in 100 μL of Prep-n-Go buffer plus 100 μL nuclease-free water. Then the samples were put in a dry bath at 90 °C during 20 min for the cellular lysis to occur.
(3) Samples were extracted with PrepFiler™ Express, and purified in the Auto Mate Express™ Forensic DNA Extraction System, according to the manufacturer's protocol [6].

### DNA quantification

Samples were quantified using Quantifiler® Trio DNA Quantification kit in an Applied Biosystems® 7500 Real-Time PCR System, according to the manufacturer's instructions [7] and were analysed using the HID Real-Time PCR Analysis Software v1.2.

### PCR amplification

All samples were amplified using PowerPlex® Fusion 6C System according to the manufacturers protocol [8]; some blood stains and saliva samples were amplified from extractions as shown in Table 1; all blood and saliva stains were directly amplified as shown in Table 2.

**Table 1.** PCR amplification protocol and PCR conditions used for amplifications of extracted samples.

PCR amplification mix	Volume per reaction
Water^a^, amplification grade	Up to 14 μL
*5X Master Mix*	5 μL
*5X Primer Mix*	5 μL
DNA^b^ (sample)	1–15 μL
Final reaction volume	25 μL

Run the recommended protocol for 29 cycles;

a Water is used to normalize the final reaction volume;

b DNA volume can be variable according to previous quantification results.

**Table 2.** PCR amplification protocol and PCR conditions used for direct amplifications.

PCR amplification mix	Volume per reaction
Water, amplification grade	5 μL
*5X Master Mix*	2.5 μL
*5X Primer Mix*	2.5 μL
*5X AmpSolution*	2.5 μL
DNA (sample)	1.2 mm per punch
Final reaction volume	12.5 μL

Run the recommended protocol for 25 cycles.

### Capillary electrophoresis and data analysis

Amplified PCR products were separated and detected in an Applied Biosystems® 3500 Genetic Analyzer using a mixture of 0.5 μL WEN ILS 500 Standard and 9.5 μL Hi-Di Formamide per sample. Samples were injected at 1.2 kV for 15 s. Electrophoresis results were analysed with GeneMapper® ID-X v1.4.

### Specificity

To ensure that the kit only amplifies human DNA, nine blood samples from different non-human animal species, four samples from *Equus caballus* (horse), three samples from *Sus scrofa domesticus* (pig) and two samples from *Canis lupus familiaris* (dog) were amplified. These samples were then extracted with PrepFiler™ Express and purified in the AutoMate Express™, followed by amplification with PowerPlex® Fusion 6C System, with the maximum volume of DNA that the kit allows (15 μL) in a final reaction volume of 25 μL. Samples were analysed with GeneMapper® ID-X software v1.4 using a 50 relative fluorescent unit (RFU) threshold.

### Analytical threshold

An AT is the lowest RFU value at which DNA can be distinguished from background noise and it must be defined prior to analysis of the samples. To do that, 16 reagent blanks containing only the mixture for separation and detection (0.5 μL WEN ILS 500 + 9.5 μL Hi-Di Formamide) were distributed in a 96-well plate, filling the first two rows of the plate.

The plate ran on a 3500 Genetic Analyzer and the injection of the samples was done in triplicate totalizing 64 reagent blanks. Electropherograms were obtained and the RFU value from the highest peak in each colour panel was introduced in an excel sheet for all the 64 blanks. The values were rearranged in ascendant order, outliers were eliminated and the rest of the values were divided in quartiles (Q). For the calculation, it was used the AT formula proposed by the Spanish and Portuguese-Speaking Working Group of the International Society for Forensic Genetics (GHEP-ISFG) [9] which is AT = Q3 + 3 × IQR. Q3 means RFU value at third quartile, and IQR means the value difference between first quartile and third quartile.

### Sensitivity

According to manufacturer's manual, the recommended DNA input for optimal results should be 1 ng. Extracted DNA samples, with different extraction methodologies, previously quantified and normalized to approximately 1 ng/μL, were serial diluted to 1:10, 1:50, 1:100 and quantified. Since the maximum volume for amplification of extracted DNA is 15 μL, for the samples that had a total DNA amount under 1 ng, the maximum volume was used, the rest of the samples were amplified with different volumes varying from 2 to 15 μL trying to reach the 1 ng input.

### Precision

To verify the precision of allele designation, 18 ladders were analysed through the process of validation.

Two of the samples analysed in the process of validation had loci with genotypes that differ only by a single base pair. One of the samples had the locus *D2S441* with the following genotypes: 11.3 and 12; the other sample had the locus *D12S391* with the following genotypes: 18.3 and 19. These samples were diluted to 1:10, 1:50 and 1:100 and amplified to demonstrate single-base resolution.

### Mixture studies

Five samples previously extracted/prepared (2 by Prep-n-Go™ – A and B; 2 by Chelex® – C and D; and 1 by PrepFiler™ – E) were combined in 10 different mixtures and in 3 different ratios (1:10, 1:1 and 10:1) totalizing 30 different mixtures.

### Volume reduction

The recommended final reaction volume for extracted DNA is 25 μL and for direct amplification is 12.5 μL. To test if the same volume for both amplifications could be used, 24 samples previously prepared with Prep-n-Go™ buffer were amplified with PowerPlex® Fusion 6C System according to manufacturer's protocol, and the same 24 samples were also amplified with the reduction of the reaction volume to 12.5 μL. The rest of the analysis was done without changes.

### Cycle number adaptations

The recommended cycle number is 29 cycles for extracted DNA samples. Besides this recommended cycle number, the same 24 samples from the previous parameter were also tested with 26 and 27 cycles to understand which one would work better. For this parameter, the final reaction volume was 12.5 μL.

### Direct amplification

Blood stains and saliva stains, totalizing 24 samples, were amplified directly without previous extraction, according to the manufacturer's protocol for direct amplification [8].

An adaptation to the preparation of the mixture for amplification was also tested by removing the reagent *5X AmpSolution*™ and instead of this buffer, the final volume was compensated with water.

Alongside with this adaptation, the reduction of the punch size from 1.2 to 0.5 mm and the increasing of cycle number from 25 to 26 cycles were also tested. The same 24 samples were amplified with these adaptations.

### DNA control samples

To guarantee the quality of the genetic profiles obtained with the kit, human genomic DNA control samples with different concentrations (9947A: 2 and 10 ng/μL; 007: 2 ng/μL; and 2 800 M: 10 ng/μL) were amplified. All the samples were normalized to 1 ng/μL and amplified with the recommended protocol.

### Proficiency test

Every year, the laboratory participates in proficiency tests organized by GHEP-ISFG, a collaborative exercise is done by several laboratories in order to discuss the results later. These samples can be used as mock evidence samples for validation purposes.

The exercise has basic and advanced levels with different types of samples. The basic one had blood and saliva stains that were then extracted by Chelex® and a piece of fabric with a mixture of sperm and saliva extracted by PrepFiler™. In the advanced one, all the samples were then extracted by PrepFiler™, it had a piece of wood with sperm, a tie with blood, a bracket brush with saliva and a piece of gauze with non-human blood and sperm. All samples were amplified with PowerPlex® Fusion 6C System.

## Results and discussion

### Specificity

No genetic profiles were obtained for the analysed samples; in two samples from *Sus scrofa domesticus* (pig) an allele was assigned in the genetic marker *SE33* (28) which is described in the literature as an artefact [3].

### Analytical threshold

From the 64 analysed samples, 4 were eliminated due to discrepant values (outliers), totalizing 60 samples. The rearranged values were analysed and the obtained values, for each colour panel, are described in Table 3.

**Table 3.** Obtained RFU values for the quartiles and interquartile amplitude from each panel and respective analytical threshold (AT).

Panel	Q1	Q3	IQR	AT
Blue	10.5	14	3.5	24.5
Green	23	31	8	55
Yellow	57	74	17	125
Red	21	27.5	6.5	47
Purple	53	66.5	13.5	107

Q1: first quartile; Q3: third quartile; IQR: interquartile amplitude.

Although the obtained values for AT of each colour panel are very different from each other, a single value should be defined to be used in every panel, so the analysis could be standardized. The highest value obtained, 125 RFU in the yellow panel, should be the value selected to be used in all the panels because a value under this could compromise allele designation in the yellow panel.

### Sensitivity

**Figure 1.**
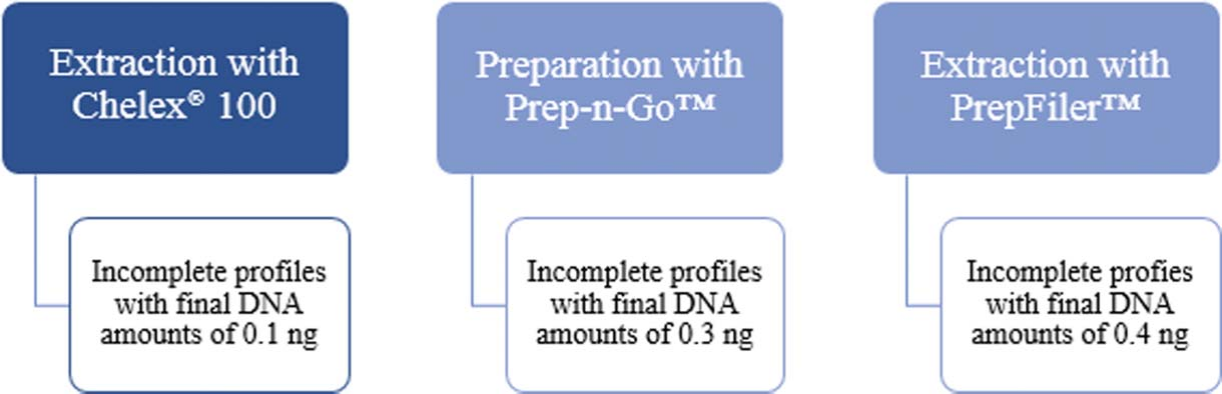
Sensitivity results for the different extraction methodologies used, Chelex® 100, Prep-n-Go™ and PrepFiler™.

Final DNA amounts, ranging from 0.09 to 1.5033 ng were obtained. Different values were obtained from the samples extracted with different methodologies; this can be justified with the fact that each method is different and each sample is unique and has its own characteristics. Results of sensitivity for different extraction methodologies are described in Figure 1. Those results were obtained with 15 s injection in a 3500 genetic analyser.

A 40 s injection was also tried and in all samples extracted with Chelex® complete profiles were obtained, with all the alleles assigned. Profiles began to be compromised at 0.1 ng with Prep-n-Go, and at 0.2 ng with PrepFiler™.

Considering all the extraction methods, a complete profile can be obtained, for the samples analysed, with quantities of DNA above 0.4 ng for a 15 s injection and 0.2 ng for a 40 s injection which is a lower value than the recommended (1 ng). Those results are in concordance with previous studies [3, 10].

### Precision

All the 18 ladders were analysed and compared between each other and it was confirmed that all the alleles were correctly assigned.

For both samples, one with the locus *D2S441*: genotypes 11.3 and 12, and the other one with the locus *D12S391*: genotypes 18.3 and 19, the alleles were correctly assigned through all the dilutions; the most diluted samples (1:100) had DNA quantities of 0.3 and 0.1 ng and could demonstrate single-base precision in the size range of PCR products and with low quantities of DNA.

### Mixture study

**Figure 2.**
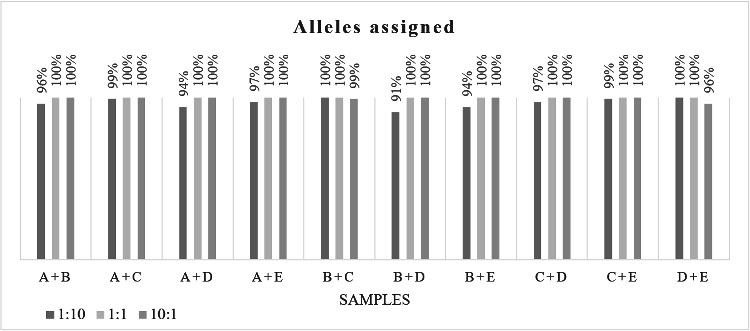
Percentage of assigned alleles in different mixtures prepared in different ratios from samples A and B (prepared with Prep-n-Go), C and D (extracted with Chelex®) and E (extracted with PrepFiler™).

Mixture profiles were detected in all the samples analysed, 20 out of 30 (66.7%) mixture profiles were complete; all mixtures with 1:1 ratio were completely identified. The rest of the profiles (33.3%) had drop-out alleles associated with the minor contributor of the mixture in 10:1 and 1:10 ratios; despite that it was possible to assign more than 90% of the alleles in those profiles (Figure 2).

### Volume reduction

**Figure 3.**
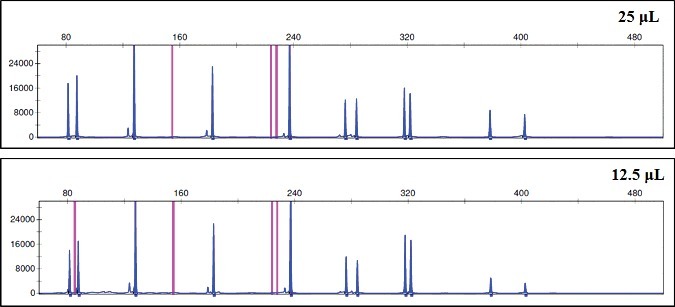
Representative electropherogram of human genomic DNA at 1 ng with both final reaction volumes tested, 25 and 12.5 μL. Scale is 32 000 RFU.

After the samples were amplified, all the electropherograms showed similar results with both reaction volumes of 12.5 and 25 μL (Figure 3). With the final reaction volume of 12.5 μL, signal increases and profiles are more balanced. No new artefacts were observed. Those results are in concordance with previous studies [3].

### Cycle number adaptations

**Figure 4.**
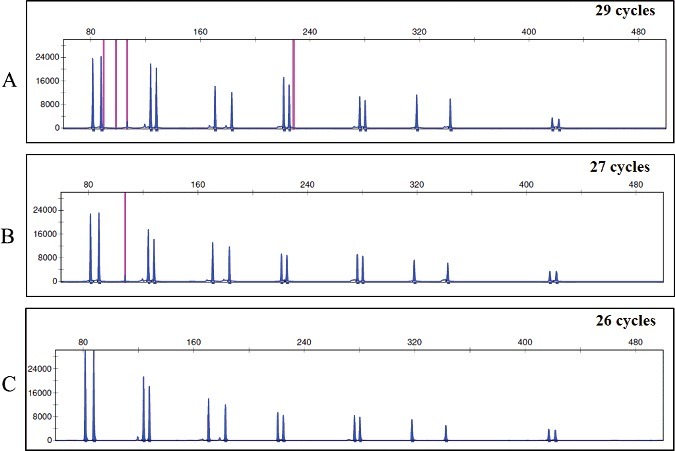
Representative electropherogram of human genomic DNA at 1 ng with different cycle number adaptation. (A) Recommended protocol: 29 cycles; (B) 27 cycles; (C) 26 cycles. Scale is 32 000 RFU.

Profiles obtained from the analysed samples with both 29 and 27 cycles were over-amplified and had some artefacts (Figure 4(A,B)). When the number of cycles was reduced to 26, the profiles were more balanced, without over-amplification; the overall signal was not reduced and showed less artefacts. With 26 cycles, all the alleles were correctly assigned with complete profiles and significant peak heights (Figure 4(C)). Those results are in concordance with previous studies [3], although for the protocol with 27 cycles no results were found in other studies.

### Direct amplification

**Figure 5.**
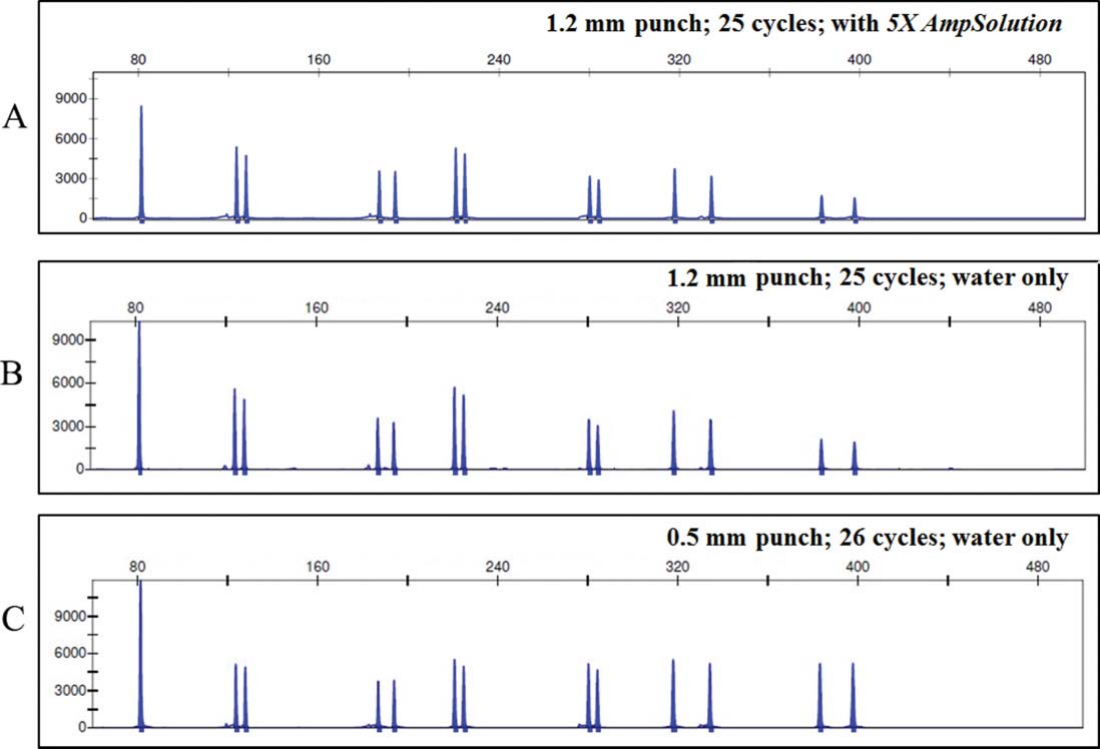
Representative electropherogram of human genomic DNA directly amplified using different protocols. (A) Recommended protocol: 1.2 mm punch, 25 cycles with the reagent *5X AmpSolution*; (B) 1.2 mm punch, 25 cycles with only water; (C) 0.5 mm punch, 26 cycles with only water. Scale is 12 000 RFU.

Direct amplification protocol was tested and led to balanced and complete profiles (Figure 5(A)).

For samples tested with water, instead of the reagent *5X AmpSolution*, there was no difference in the amplification. Removing the *5X AmpSolution* reagent from the amplification mixture produced no effect on the signal or on the balance of the profile (Figure 5(A,B)). Lowering the punch size from 1.2 to 0.5 mm and increasing the number of cycles from 25 to 26 allowed the simultaneous amplification of extracted and direct samples with the same thermocycler protocol. These adaptations enabled the obtainment of equally good profiles (Figure 5(B,C)). Those results are in concordance with previous studies, although for the protocol where only water is used, no results were found from other studies.

### DNA control samples

**Figure 6.**
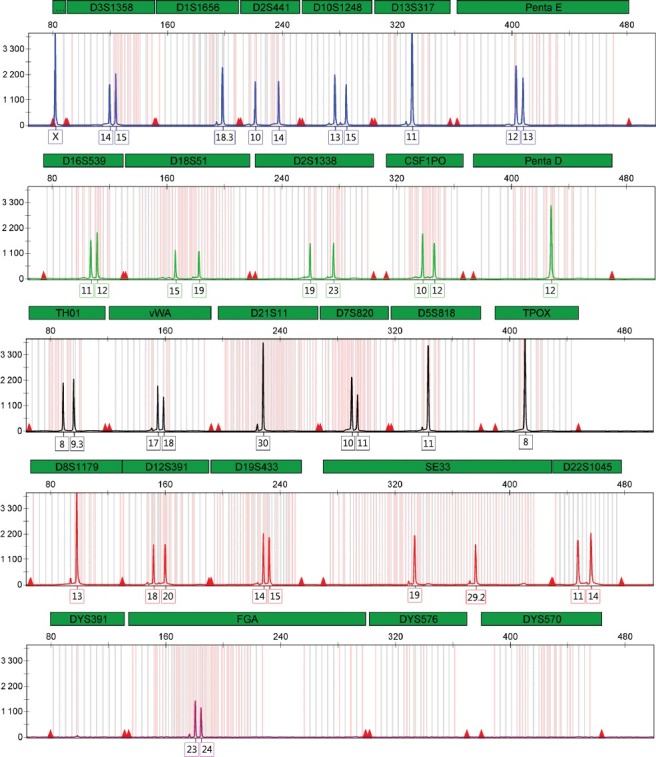
Amplification of 1 ng 9947A DNA control sample with PowerPlex® Fusion 6C System following the recommended protocol. Panel labelled “…” is Amelogenin. Scale is 4 400 RFU.

A complete and balanced genetic profile was obtained from all the samples analysed; the quality of the profiles obtained was the expected considering that the samples are stable and were amplified with the recommended quantity of DNA (1 ng). An example of a control DNA amplified with PowerPlex® Fusion 6C System is shown in Figure 6.

### Proficiency test

All of the samples, from both basic and advanced exercises gave results concordant with the consensus result presented by GHEP-ISFG.

## Conclusion

PowerPlex® Fusion 6C System proved to be a very robust kit, enabling balanced and complete profiles with 26, 27 and 29 cycles. Reducing the punch size, increasing cycle number and removing the *5X AmpSolution* reagent in direct amplification protocol revealed equally good results as with the original protocol.

No incompatibility was found with methodologies, equipments, storage conditions and workflow of the Genetic Service. The study with samples from non-human DNA ensured that this kit is specific to amplify human DNA. Based on reagent blanks the AT to be used was defined as 125 RFU. Complete profiles were obtained from samples with only 0.4 ng of DNA which is much lower than the manufacturers’ recommendations (1 ng). Precision studies revealed strong capacity to assign alleles that differ in a single base pair even in samples with low quantities of DNA (0.1 ng). Using PowerPlex® Fusion 6C System was possible to identify without any doubt a mixture profile; most of the profiles were complete (66.7%) and the incomplete ones had more than 90% of the alleles assigned.

All the changes done to the original procedure did not compromise the ability of the kit to obtain good results. Reference samples often have high amounts and quality of DNA so that the adaptations can be used with those samples, simplifying the methodology for both extracted samples and direct amplifications, using less reagents and saving some time.
